# High β-carotene accumulation in transgenic eggplant fruits grown under artificial light

**DOI:** 10.5511/plantbiotechnology.23.1129b

**Published:** 2024-03-25

**Authors:** Ryohei Yamamoto, Seigo Higuchi, Yuji Iwata, Satomi Takeda, Nozomu Koizumi, Kei-ichiro Mishiba

**Affiliations:** 1 Faculty of Agriculture, Ryukoku University, 1-5 Yokotani, Seta Oe-cho, Otsu, Shiga 520-2194, Japan; 2 Graduate School of Agriculture, Osaka Metropolitan University, 1-1 Gakuen-cho, Nakaku, Sakai, Osaka 599-8531, Japan; 3 Graduate School of Sciences, Osaka Metropolitan University, 1-1 Gakuen-cho, Nakaku, Sakai, Osaka 599-8531, Japan

**Keywords:** β-carotene, eggplant, fruit, genetic engineering

## Abstract

Eggplant (*Solanum melongena* L.) fruits are known to contain few carotenoids such as β-carotene, which are abundant in congener tomato fruits. In a previous study, we introduced a fruit-specific *EEF48* gene promoter-driven *crtB* gene encoding phytoene synthase (PSY) of *Erwinia uredovora* into eggplant ‘Senryo No. 2’. The transgenic plants grown in a greenhouse set fruits that accumulated β-carotene (∼1.67 µg g^−1^FW) in the T_0_ and T_1_ generations. In the present study, we grew T_1_ and T_2_ generations of the transgenic eggplant plants in artificial climate chambers to investigate their fruit set and β-carotene accumulation. No clear difference in β-carotene accumulation was observed in the fruit of transgenic plants grown under either HID (high-intensity discharge) or LED (light-emitting diode) light, or between T_1_ and T_2_ generations. The β-carotene accumulation (8.83 µg g^−1^FW on average) was approximately 5 times higher than the previous results obtained from greenhouse-grown plants. However, the fruit weight and size of the T-DNA (+) plants were significantly smaller than that of their null-segregant T-DNA (−) plants derived from the same line, suggesting that β-carotene accumulation may inhibit fruit development. Considering that a part of plants grown under LED irradiation failed to set fruits or set smaller fruits than those grown under HID irradiation, the light condition in the LED chamber may not be sufficient to promote fruit development. The present results are expected to provide valuable information for the selection of transgenic eggplants with high β-carotene content in fruit under artificial lighting.

Eggplant (*Solanum melongena* L.) is a crop cultivated globally, especially in Asia (Weese and Bohs 2010), and is an important source of nutrients for low-income consumers in developing countries (Gürbüz et al. 2018), with high fiber, potassium, and antioxidant content (San José et al. 2014). However, compared to other solanaceous crops such as tomatoes and peppers, eggplant fruits have a low provitamin A carotenoid content (Gürbüz et al. 2018). Vitamin A deficiency is a serious problem in many areas where eggplants are cultivated (Rahman et al. 2017), leading to various health problems such as night blindness, total blindness, and irreparable blindness (Dowling and Wald 1958). To address the problems through metabolic engineering, we have developed transgenic eggplant that produces fruit accumulating β-carotene by modifying the carotenoid biosynthetic pathway (Mishiba et al. 2020). In this study, a fusion gene comprising the *EEF48* gene promoter (Nagasawa et al. 2001), which is expressed specifically in eggplant fruit (Mishiba et al. 2020), and the *crtB* gene (Misawa et al. 1990) encoding a phytoene synthase (PSY) from the bacterium *Erwinia uredovora*, was introduced into eggplant ‘Senryo No. 2’ (Takii Seed, Kyoto, Japan). The transgenic T_0_ and T_1_ plants grown in a greenhouse set fruits that accumulated ∼1.67 µg g^−1^FW (fresh weight) of β-carotene (Mishiba et al. 2020). In addition to eggplant, metabolic engineering of β-carotene has been carried out, most notably in the well-known ‘Golden Rice’. This is a recombinant rice plant in which β-carotene is accumulated in the endosperm by introducing a *psy* from trumpet daffodil and a phytoene desaturase (*crtI*) gene from *Erwinia uredovora*, resulting in the accumulation of around 1.6 µg g^−1^DW (dry weight) of carotenoids (mainly β-carotene) of edible part (Ye et al. 2000). Subsequently, ‘Golden Rice 2’ was produced by introducing the maize *psy1* gene instead of the trumpet daffodil *psy* gene, increasing β-carotene accumulation to around 31 µg g^−1^DW (Paine et al. 2005). ‘Golden Rice 2’ has been approved for commercial cultivation in the Philippines in 2021. Compared with the accumulation of β-carotene in the endosperm of the ‘Golden Rice 2’, the accumulation of β-carotene in the transgenic eggplant fruits has not been sufficient to alleviate vitamin A deficiency (Mishiba et al. 2020).

Despite Japan is one of the world’s leading importers of genetically modified (GM) crops, no commercial cultivation of GM crops occurs in an open environment, except for ornamental plants (Turnbull et al. 2021). One of the barriers to achieving GM cultivation in an open environment in Japan is the requirement for a biological diversity risk assessment report. Consequently, cultivating GM crops in closed systems, such as plant factories, may provide a solution (Goto 2012). For example, research has been conducted on the cultivation of transgenic tomatoes that accumulated recombinant miraculin protein in fruits in a plant factory (Kato et al. 2011). So far, there have been reports of eggplant transplants being cultivated in a closed plant factory with artificial lighting (Yang et al. 2023). However, to our knowledge, there have been no studies investigating fruit development in eggplant under artificial lighting. In this study, we investigated the feasibility of eggplant fruit setting in a closed environment. Furthermore, we examined the effect of artificial light on the development and β-carotene content in fruit of the EEF48-*crtB* transgenic eggplant, which was developed in our previous study (Mishiba et al. 2020).

The transgenic plants used in this study are depicted in Supplementary Figure S1. T_1_ seeds were harvested from fruits of the EEF48-*crtB* plant line #2 (T_0_), which contained a single-copy T-DNA, obtained from self-fertilization or cross-pollination with pollen from EEF48-*GUS* plant line #2 (T_0_) (Mishiba et al. 2020). T_2_ seeds were harvested from self-pollination of the T_1_ EEF48-*crtB* line #2-19 (with T-DNA) and null-segregant T_1_ line #2-17 (without T-DNA; Mishiba et al. 2020). The harvested seeds were stored in sealed containers with silica gel in a refrigerator at 4°C until used. The seeds were treated with 100 mg l^−1^ gibberellin for 2 h at room temperature, and surface-sterilized with 70% ethanol for 30 s followed by sodium hypochlorite solution containing 1% active chlorine and 0.05% Tween-20 for 15 min. The sterilized seeds were rinsed three times with sterile water and sown on MS plate medium (Murashige and Skoog 1962) with 3% sucrose and 0.8% agar (pH 5.8) in a plastic petri dish (φ90 mm×20 mm). The culture conditions were 22°C in the dark for 7 days followed by a 16-h photoperiod (35 µmol m^−2^ s^−1^). After germination, seedlings were transferred to the same medium in a plant box (60 mm×60 mm×100 mm; CUL-JAR300; Iwaki, Tokyo, Japan) and cultured for 3 to 4 weeks. The in vitro grown plants were acclimatized for at 22°C with a 16-h photoperiod (35 µmol m^−2^ s^−1^). Plants were then transferred to pots (φ190 mm×150 mm) filled with sterilized culture soil (horticultural culture soil and vermiculite mixed at a ratio of 1 : 2 and sterilized by autoclaving) at 25°C with a 16-h photoperiod [330 µmol m^−2^ s^−1^ with HID (high-intensity discharge) lighting or 220 µmol m^−2^ s^−1^ with LED (light-emitting diode) lighting]. In the HID growth chamber, HID lamps (1,000 W metal halide lamp) were mounted on the ceiling of the chamber at 2.0 m height (Figure 1A). In the LED growth chamber, LED lamps (20 W white LED) were attached to cultivation lacks at a height of 0.7 m (Figure 1C). The spectral power distributions of the HID and LED chambers were measured with an illuminance meter (CL-70F, Konica Minolta, Tokyo, Japan; Supplementary Figure S2). To confirm the EEF48-*crtB* T-DNA insertion into the plants analyzed, genomic DNA was extracted (Kasajima et al. 2004) from leaves and genome PCR was performed using KOD FX Neo DNA Polymerase (Toyobo, Osaka, Japan) according to the manufacturer’s instructions. For amplification of a 304-bp fragment of the *crtB* gene, following primers were used: 5′-ATA CGC TGC GCT ATT GCT ATC ACG TT-3′ and 5′-GTG GCA GAC AAA TAG TAA GGT TCT GCT TCC-3′. RNA extraction and reverse transcription quantitative PCR (qRT-PCR) was performed as previously described (Mishiba et al. 2019). The primer sequences for *crtB* and *actin* (FS070124) genes were: (*crtB*) 5′-ACT GAT GCT CTA CGC CTG GT-3′ and 5′-GCC ATA GCC ACT TCC TGA AA-3′, (*actin*) 5′-TGT TGG ACT CTG GTG ATG GTG-3′ and 5′-TGA ATG AGT AAC CAC GCT CAG TG-3′, respectively. For HPLC analysis, frozen fruit was ground in liquid nitrogen and 0.5 g of the powder was placed in a tube and extracted by sonication for 2 min with 1 ml of ice-cooled 85% acetone in 10 mM Tris-HCl (pH 7.4). After centrifuging the mixture at 10,000×g for 60 s, the supernatant was transferred to an ice-cold test tube. The residue was re-extracted with acetone until the pellets became completely colorless. A total of 3 or 4 ml of extracts was filtered through a PTFE syringe filter with a pore size of 0.22 µm (Shoko Science, Yokohama, Japan) and immediately subjected to HPLC analysis. The extracted pigments were separated by reverse phase HPLC. The samples were applied on a Cosmosil 5C18 MS-II column (250×4.6 mm, particle size: 5 µm; Nacalai tesque, Kyoto, Japan) using a mobile phase consisting of acetonitrile : ethanol in the ratio of 70 : 30 (v/v) at a flow rate of 1.2 ml min^−1^ at a column temperature of 35°C. Chromatograms were monitored at a wavelength of 450 nm using a photodiode array detector (Jasco, Tokyo, Japan). From pigment extraction to sample application to HPLC, all experiments were performed under dim light conditions. A standard of β-carotene was purchased from Nacalai tesque. Statistical analyses were performed using EZR software (Kanda 2013).

**Figure 1:**
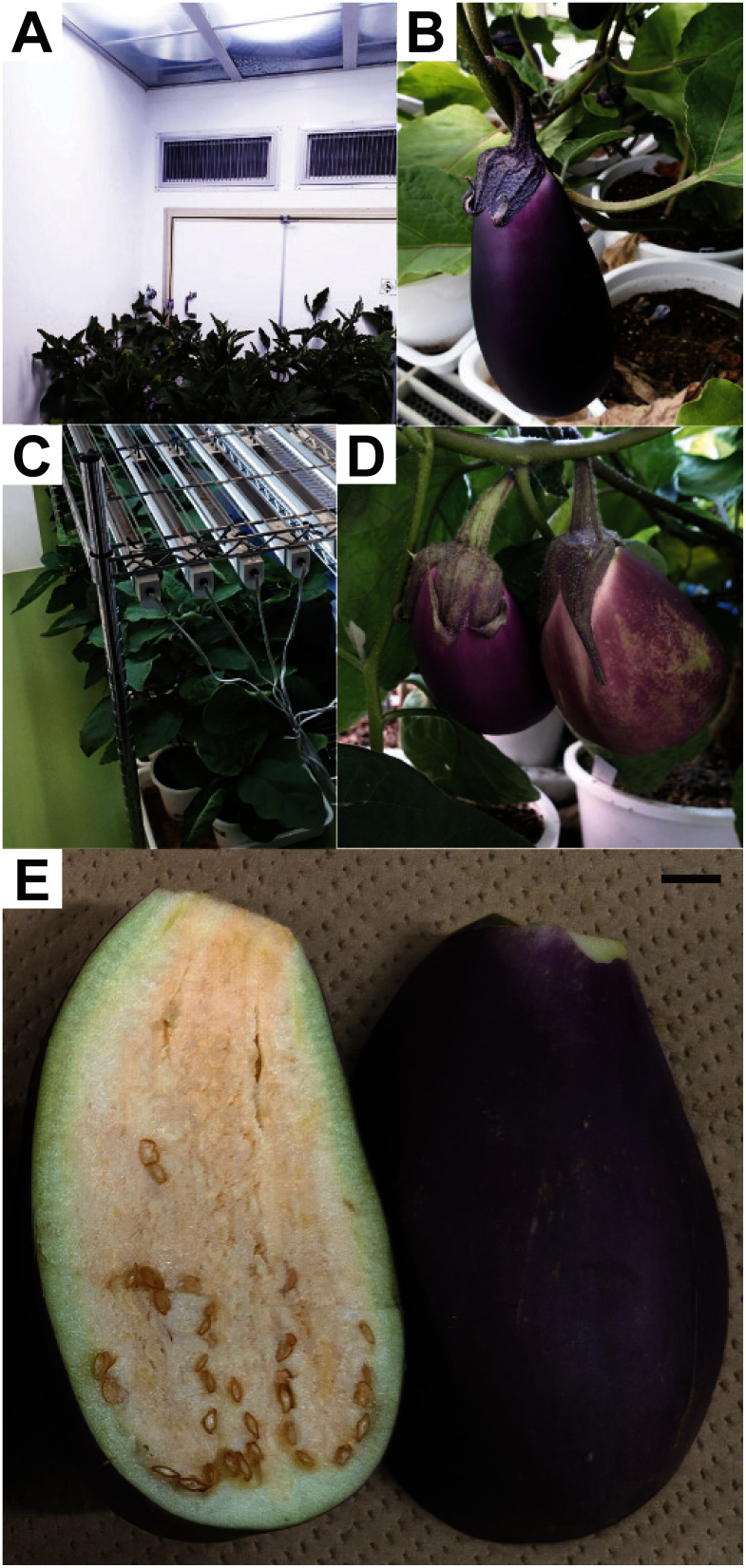
Figure 1. Cultivation of the T_0_ and T_1_ EEF48-*crtB* transgenic line #2-derived progenies in closed chambers. Acclimatized plants (A) and fruit (B) were grown in the HID chamber. Acclimatized plants (C) and fruit (D) were grown in the LED chamber. (E) Longitudinal section of the fruits derived from the EEF48-*crtB* #2-derived self-pollinated T-DNA (+) T_1_ plant. Bar=10 mm.

Sterilized T_1_ and T_2_ seeds were germinated and grown on MS plates. The T-DNA insertion of the plants was confirmed by genome PCR for the *crtB* gene. The in vitro cultured plants were acclimatized and transplanted in the HID (Figure 1A) and LED (Figure 1C) growth chambers. Most of the plants set fruit after self-pollination under HID irradiation (Figure 1B), while a part of the plants either failed to set fruit or set small fruit under LED irradiation (Figure 1D). Fruits were harvested at the stage of marketable maturity and their weight, size, and β-carotene content were determined. For the T_1_ T-DNA (−) (null-segregant) plants, fruits were obtained from one self-fertilized plant under HID irradiation, and two cross-pollinated (×EEF48-*GUS*) plants under LED irradiation [note that T-DNA (−) does not indicate the absence of the EEF48-*GUS* containing T-DNA]. For the T_1_ T-DNA (+) lines, fruits were obtained from two self-fertilized and two cross-pollinated plants under HID irradiation, and two out of three self-fertilized and one out of two cross-pollinated plants under LED irradiation. For the T_2_ T-DNA (−) lines, fruits were obtained from two #2-17- and two #2-19-derived plants under HID irradiation, and from three out of five #2-17-derived plants under LED irradiation. For the T_2_ T-DNA (+) lines, fruits were obtained from two #2-19-derived plants under HID irradiation, but no fruit was set on one #2-19-derived plant grown under LED irradiation. In the T_2_ T-DNA (−) plants grown under HID irradiation, no significant difference in fruit weight and size was observed between #2-17- and #2-19-derived plants (Figure 2A–C). This suggests that the selfed progenies of the #2 (T_0_)-derived two T_1_ lines have low genetic variation with respect to fruit size. On the other hand, the fruit length of the null-segregant #2-17-derived T_2_ plants grown under LED irradiation was significantly shorter than that of the same #2-17-derived T_2_ plants grown under HID irradiation (Figure 2F), suggesting that the HID light condition is more suitable for eggplant fruit development than the LED light condition. A previous study demonstrated that the weight of eggplant fruits grown in a greenhouse was reduced under conditions of ultraviolet- and far-red-liming light by the use of energy-saving film (Chavan et al. 2020). This may partially explain the inferior fruit growth observed under the LED light spectrum (Supplementary Figure S2). Fruits obtained from the T-DNA (+) plants accumulated β-carotene regardless of the light conditions (Figure 1E). In the #2-19-derived plants grown under HID irradiation, the average β-carotene content of fruits from the T-DNA (+) plants was 9.42 µg g^−1^FW, whereas no β-carotene could be detected in fruits obtained from the T-DNA (−) plants (Figure 2D). There was no clear correlation between pericarp color and β-carotene accumulation in fruit. The two #2-19-derived T-DNA (+) plants grown under HID irradiation, designated as #2-19-1 and #2-19-3, exhibited homo- and hemizygous for T-DNA insertions, respectively (Supplementary Figure S3A). Consistently, *crtB* mRNA expressions in leaf and fruit in the homozygous #2-19-1 were higher than those in the hemizygous #2-19-3 (Supplementary Figure S3B). However, there was no significant difference in β-carotene content in fruit between the #2-19-1 and #2-19-3 plants (data not shown). This suggests that *crtB* mRNA expression in these transgenic plants may not be a limiting factor for β-carotene accumulation in fruit. The fruit weight and size of the #2-19-derived T-DNA (+) plants were significantly smaller than those of the #2-19-derived T-DNA (−) plants (Figure 2A–C). Since ectopic expression of the phytoene synthase gene caused abnormal growth in transgenic tomato and tobacco (Busch et al. 2002; Fray et al. 1995), it is possible that ectopic expression of the phytoene synthase gene (*crtB*) may adversely affect eggplant fruit development. In the T-DNA (+) plants grown under HID irradiation, there was no significant difference in fruit weight, size, and β-carotene content among the self-fertilized and cross-pollinated T_1_ plants and #2-19-derived T_2_ plants (Supplementary Figure S4A–D). Consequently, we compared the fruit weight, size, and β-carotene content in the #2-derived T_1_ and T_2_ plants grown under HID irradiation with those under LED irradiation. The average β-carotene contents in fruits of the T-DNA (+) plants under HID and LED irradiation were 8.71 and 9.31 µg g^−1^FW, respectively (Figure 3D; note that LED data consist of T_1_ plants). Like the results from #2-19-derived T_2_ plants, the fruit weight and size of the T-DNA (+) plants tended to be smaller than those of the T-DNA (−) plants regardless of the light conditions, even though statistical significance was not reached (Figure 3A–C). This trend is more pronounced in the #2-derived plants grown under LED irradiation (Figure 3A–C), while β-carotene contents was not significantly different between the HID- and LED-grown plants.

**Figure 2:**
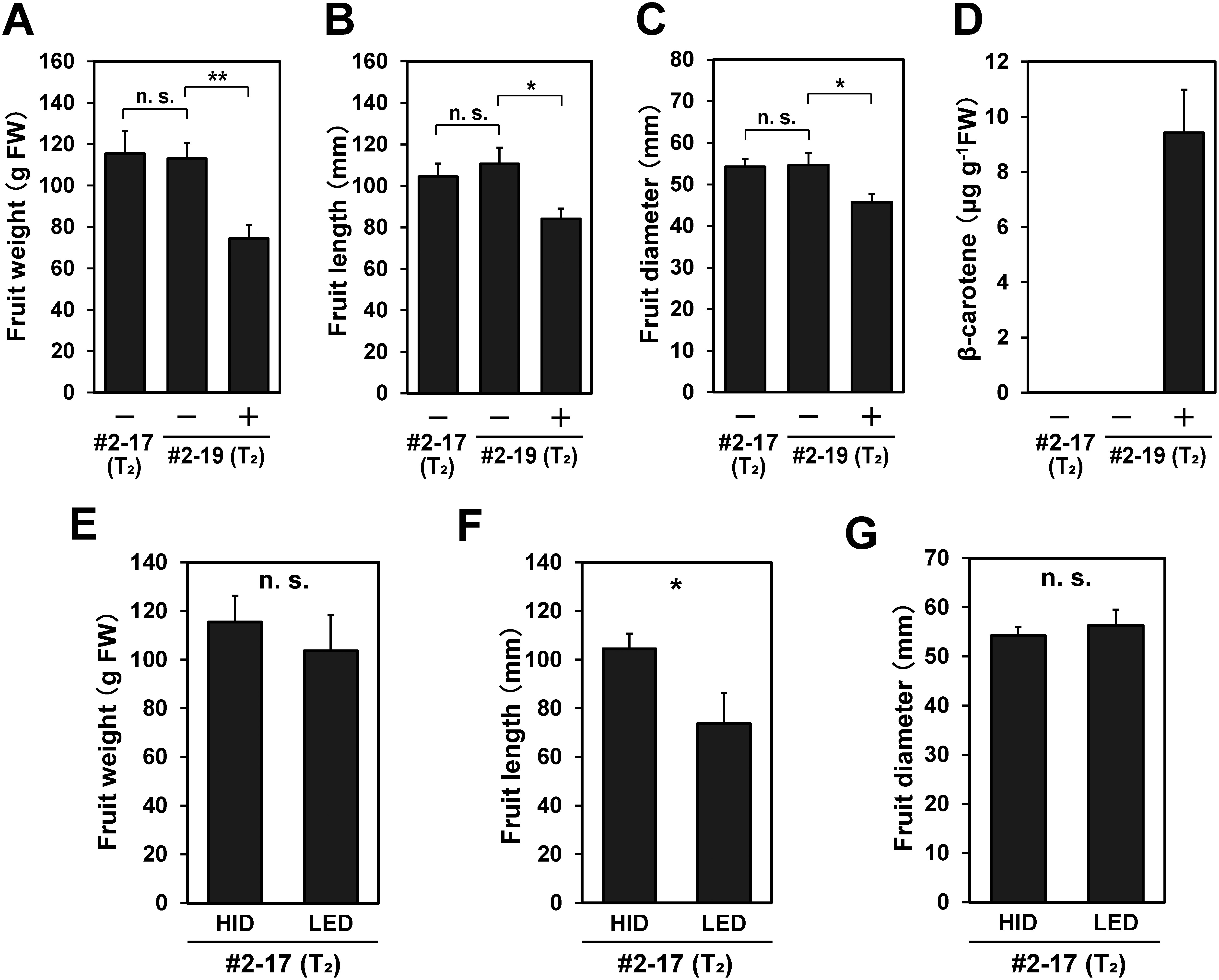
Figure 2. Fruit size and β-carotene content of T_2_ T-DNA (−) (null-segregant) and T-DNA (+) eggplant plants. The fruit weight (A, E), fruit length (B, F), fruit diameter (C, G), and β-carotene content (D) were obtained from #2-17- and #2-19-derived plants grown in the HID chamber (A–D), or #2-17-derived T-DNA (−) plants grown in the LED and HID chambers (E–G). Vertical bars represent±SE. n. s., *, and ** indicate not significant and significant at the *p*<0.05 and *p*<0.01 levels in *t*-test, respectively.

**Figure 3:**
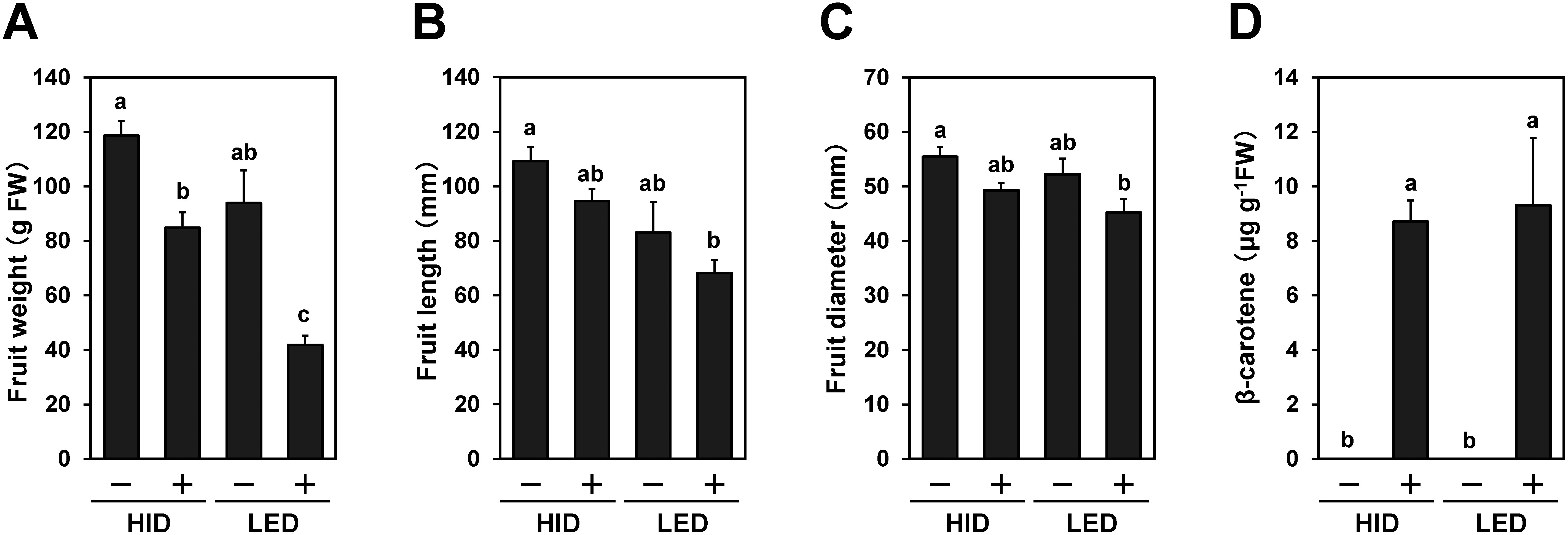
Figure 3. Size and β-carotene content of the fruits of the EEF48-*crtB* #2-derived T-DNA (−) and T-DNA (+) plants grown in the HID and LED chambers. The fruit weight (A), fruit length (B), fruit diameter (C), and β-carotene content (D) were obtained from #2-derived T_1_ and T_2_ plants grown in the LED and HID chambers. Vertical bars represent±SE. Different letters within each treatment indicate significant differences (*p*<0.05) by the Tukey–Kramer HSD test.

The β-carotene accumulation in the fruit in the present study using HID and LED (8.83 µg g^−1^FW on average) was approximately 5 times higher than in the previous study (∼1.67 µg g^−1^FW) using greenhouse-grown plants (Mishiba et al. 2020) and was comparable to the β-carotene content found in tomato fruits (∼7.8 µg g^−1^FW; Baranska et al. 2006). Considering that both of our studies used T_0_ line #2-derived self-fertilized T_1_ siblings, the most important difference between experiments appears to be the light environment. In the study of the recombinant miraculin-producing transgenic tomato fruits, a lower photosynthetic photon flux (PPF) produced a higher concentration of miraculin in fruit, even though the low PPF (100 µmol m^−2^ s^−1^) resulted in low fruit production (Kato et al. 2011). Although the transgenes and plants used in this study differ from ours, there may be some factors that promote the accumulation of recombinant proteins in fruits at low light levels. Given that the recipient cultivar ‘Senryo No. 2’ is an F_1_ variety, it is also necessary to consider the segregation of traits that occurred in the progeny. We cannot dismiss the possibility that these traits may influence the β-carotene content, and we aim to select the progeny with high β-carotene content in fruit as well as suitable traits in future selections. Although the underlying cause of β-carotene accumulation in fruits under artificial light remains elusive, we have demonstrated the feasibility of producing β-carotene-accumulating eggplants and their progeny in an artificial climate chamber. The findings of this study may contribute to improving the production of transgenic eggplant fruits with high β-carotene content.

## Abbreviations

crtB: bacterial phytoene synthase
*EEF*: eggplant early fruit
GUS: β-glucuronidase
HPLC: high-pressure liquid chromatography

## Description of Supplementary Files

Supplementary Figure S1. Schematic diagram of the transgenic plants used in the present study.

Supplementary Figure S2. Spectral power distributions of the HID (A) and LED (B) chambers.

Supplementary Figure S3. Characterization of the #2-19-derived T_2_ plants.

Supplementary Figure S4. Size and β-carotene content of the fruits of the EEF48-*crtB* #2-derived T-DNA (+) plants grown in the HID chamber.
