# Supplementary material for: High β-carotene accumulation in transgenic eggplant fruits grown under artificial light
Source: Plant Biotechnol (Tokyo). 2024 Mar 25;41(1):77–81. doi: 10.5511/plantbiotechnology.23.1129b (PMC11500568; doi:10.5511/plantbiotechnology.23.1129b)
Supplement: Supplementary Data [file plantbiotechnology-41-1-23.1129b-s001.pdf]

High β-carotene accumulation in transgenic eggplant fruits grown under artificial light

Ryohei Yamamoto, Seigo Higuchi, Yuji Iwata, Satomi Takeda, Nozomu Koizumi, Kei-ichiro Mishiba

Supplementary Figures

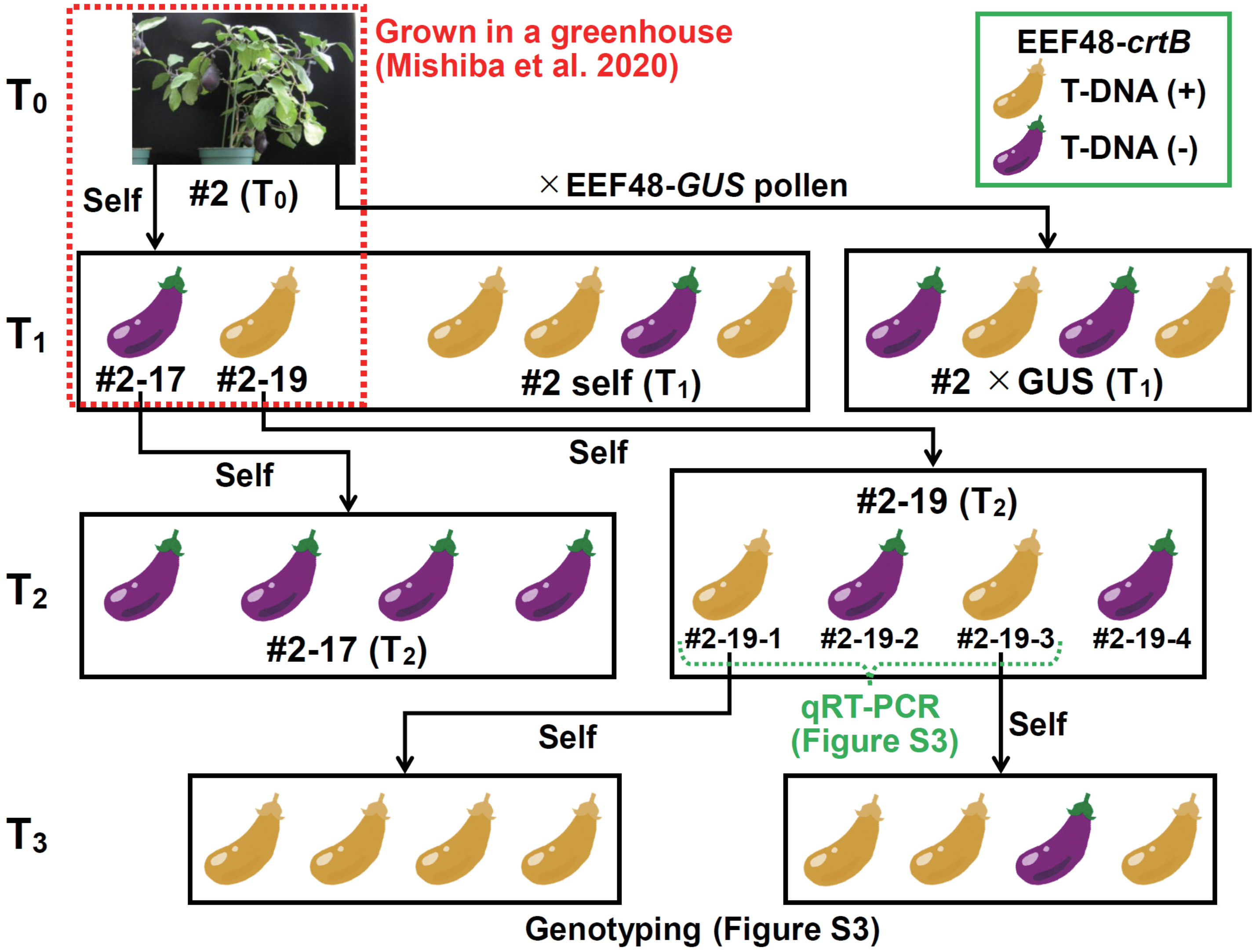

Supplementary Figure S1. Schematic diagram of the transgenic plants used in the present study. EEF48-*crtB* transgenic plants and null-segregant plants are depicted as yellow and purple symbols of eggplant fruits, respectively. Note that the T_0 #2, and T_1 #2-17 and #2-19 plants were used in the previous study (Mishiba et al. 2020) and were grown in a greenhouse (indicated by red dotted line).

Mishiba KI, Nishida K, Inoue N, Fujiwara T, Teranishi S, Iwata Y, Takeda S, Koizumi N (2020) Genetic engineering of eggplant accumulating *β*-carotene in fruit. *Plant Cell Reports* 39: 1029–1039

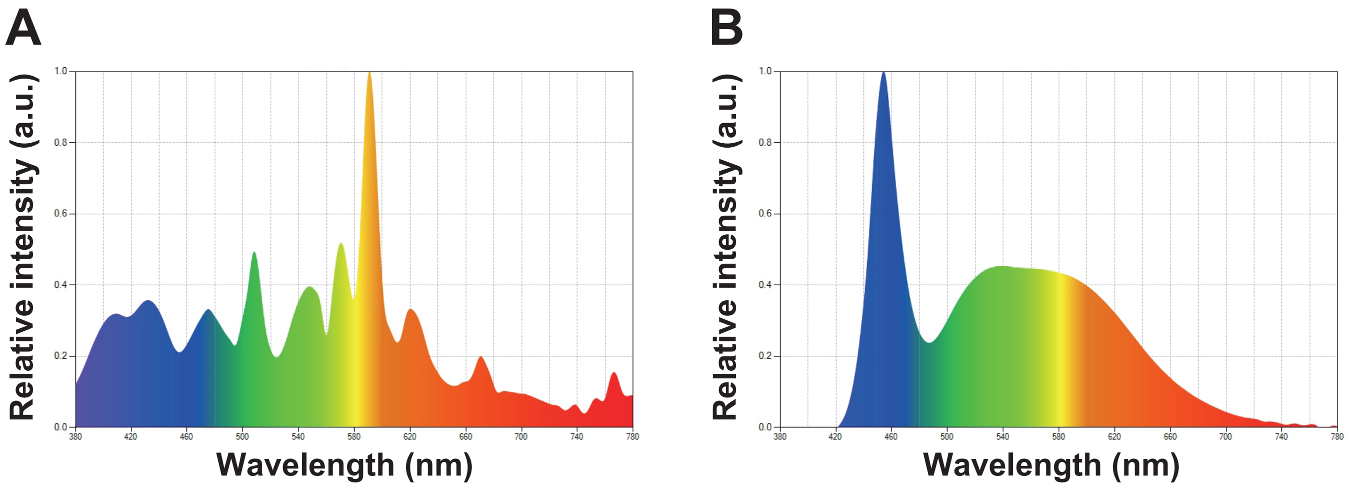

Supplementary Figure S2.   Spectral power distributions of the HID (A) and LED (B) chambers.

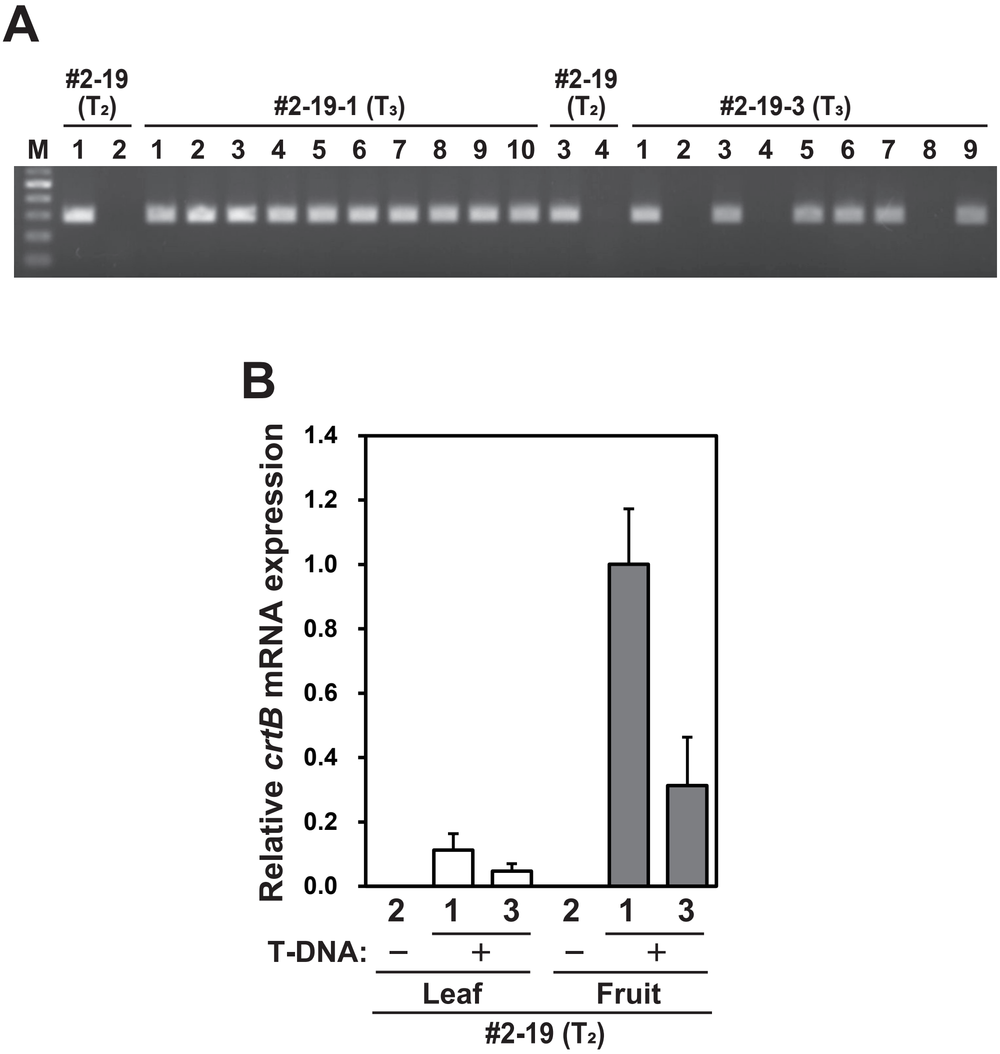

Supplementary Figure S3. Characterization of the #2-19-derived T_2 plants. (A) Genomic PCR analysis for the presence of *crtB* gene in the #2-19-derived T_2 plants, and selfed progenies of the #2-19-derived T_2 T-DNA (+) lines, #2-19-1 and #2-19-3. Lane M, 100-bp DNA ladder. (B) The relative mRNA level of *crtB* in the #2-19-derived T_2 T-DNA (−) line #2-19-2, and T_2 T-DNA (+) lines, #2-19-1 and #2-19-3. Total RNA was isolated from young leaves and fruits and subjected to reverse transcription quantitative PCR (qRT-PCR). *Actin* was used as an internal standard. The expression level in the fruit of #2-19-1 was set as 1. Data are means ± SE of three independent biological replicates.

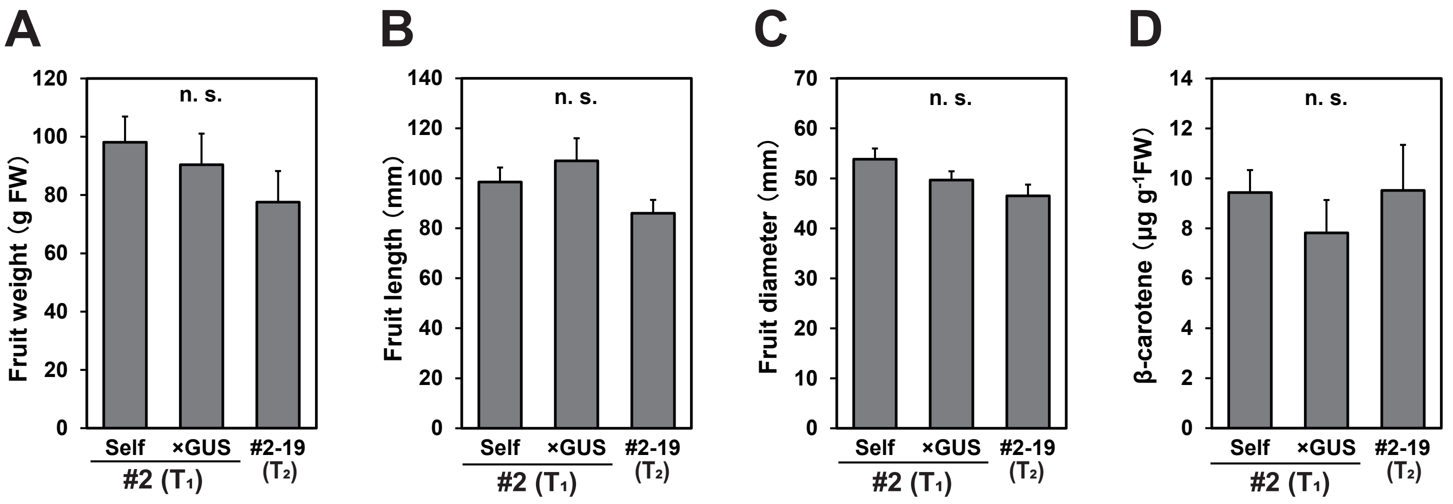

Supplementary Figure S4. Size and β-carotene content of the fruits of the EEF48-*crtB* #2-derived T-DNA (+) plants grown in the HID chamber. The fruit weight (A), fruit length (B), fruit diameter (C), and β-carotene content (D) were obtained from #2-derived T_1 self-fertilized and cross-pollinated (× EEF48-*GUS*) plants [note that T-DNA (+) does not indicate the presence of the EEF48-*GUS* containing T-DNA] and #2-19-derived T_2 plants grown in the HID chamber. Vertical bars represent ± SE. n. s. indicates no significant difference ($P > 0.05$) by the Tukey–Kramer HSD test.
